# “Preliminary Seroepidemiological survey of dengue infections in Pakistan, 2009-2014”

**DOI:** 10.1186/s40249-017-0258-6

**Published:** 2017-03-09

**Authors:** Muhammad Suleman, Hyeong-Woo Lee, Syed Sohail Zahoor Zaidi, Muhammad Masroor Alam, Nadia Nisar, Uzma Bashir Aamir, Salmaan Sharif, Shahzad Shaukat, Adnan Khurshid, Mehar Angez, Massab Umair, Ghulam Mujtaba, Rani Faryal

**Affiliations:** 1 Department of Microbiology, Quaid-i-Azam University, Islamabad, Pakistan; 2 Department of Virology, National Institute of Health, Islamabad, Pakistan; 3 Department of Tropical Medicine and Parasitology, Inha University School of Medicine, Incheon, 22212 Republic of Korea

**Keywords:** Dengue Virus, epidemiology, Pakistan, IgM ELISA, Vector

## Abstract

**Background:**

Dengue virus is the causative agent of dengue fever, a vector borne infection which causes self-limiting to life threatening disease in humans. A sero-epidemiological study was conducted to understand the current epidemiology of dengue virus in Pakistan which is now known as a dengue endemic country after its first reported outbreak in 1994.

**Methods:**

To investigate the prevalence of dengue virus in Pakistan during 2009-2014, a total of 9,493 blood samples were screened for the detection of anti-dengue IgM antibodies using ELISA. Clinical and demographic features available with hospital records were reviewed to ascertain mortalities related to dengue hemorrhagic shock syndrome.

**Results:**

Out of 9,493 samples tested, 37% (3,504) were found positive for anti-dengue IgM antibodies. Of the seropositive cases, 73.6% (2,578/3,504) were male and 26.4% (926/3,504) were female. The highest number (382/929; 41.1%) of sero-positive cases was observed among the individuals of age group 31-40 years. The highest number of symptomatic cases was reported in October (46%; 4,400/9,493), and the highest number of sero-positive cases among symptomatic cases was observed in November (45.7%; 806/1,764). Mean annual patient incidence (MAPI) during 2009-2014 in Pakistan remained 0.30 with the highest annual patient incidence (11.03) found in Islamabad. According to the available medical case record, 472 dengue related deaths were reported during 2009-2014.

**Conclusion:**

The data from earlier reports in Pakistan described the dengue virus incidence from limited areas of the country. Our findings are important considering the testing of clinical samples at a larger scale covering patients of vast geographical regions and warrants timely implementation of dengue vector surveillance and control programs.

**Trial registration number:**

It is an epidemiological research study, so trial registration is not required.

**Electronic supplementary material:**

The online version of this article (doi:10.1186/s40249-017-0258-6) contains supplementary material, which is available to authorized users.

## Multilingual abstracts

Please see Additional file 1 for translations of the abstract into six official working languages of the United Nations.

## Background

Dengue virus belongs to the genus *Flavivirus* within the family Flaviviridae*,* and contains single stranded positive sense RNA genome. There are four serotypes of dengue virus DENV1-4 [1]. The primary infection caused by a single DENV serotype is usually mild and self-limiting. The more severe form of dengue infection is dengue hemorrhagic fever (DHF) and dengue shock syndrome (DSS) responsible for high morbidity and mortality [2]. Since the introduction of new dengue guidelines by World Health Organization (WHO) in 2009, dengue is classified into three clinical stages: dengue without warning signs; dengue with warning signs (abdominal pain, persistent vomiting, fluid accumulation, mucosal bleeding, lethargy, liver enlargement, increasing hematocrit with decreasing platelets) and severe dengue (dengue with severe plasma leakage, severe bleeding or organ failure) [3].

Mostly Dengue virus infection is characterized by high grade fever, headache, skin rash, muscle pain, joint pain, and retro-orbital pain etc [4]. Typically, the secondary dengue infection with a different serotype than the primary infection is associated with severe clinical manifestations. Dengue virus is transmitted by mosquitoes mainly *Aedes aegypti* and to lesser extent by *Aedes albopictus.* The *Ae. aegypti* is a day biting mosquito and breeds in artificial as well as the natural water resources [5]. According to WHO, more than 124 countries are at risk of dengue virus infection and more than 100 million cases of dengue virus infection with 25 000 estimated deaths are reported per annum globally [6].

Pakistan reported its first outbreak of dengue hemorrhagic fever in 1994 [7]. With several subsequent outbreaks across country reported to date [8]. For instance, during 2010–2011, dengue outbreak had occurred in many districts of Punjab province including Lahore, Sheikhupura, Gujranwala, Faisalabad, Attock and Rawalpindi followed by 2013–2014 outbreaks in Swat and Mansehra [9–19].

## Methods

### Ethical approval

This study was approved by the Internal Review Board of National Institute of Health, (NIH) Islamabad, Pakistan.

### Study design

This research study was conducted on the serum samples received at Department of Virology, NIH, Islamabad Pakistan from different hospitals across the country during January 2009-December 2014. The NIH Islamabad serves as the country’s epic public health institute and receives thousands of samples for diagnostic services and disease outbreak investigations from all over the country. Due to lack of an active surveillance system and poor diagnostic capacities for hemorrhagic fever infections, samples from suspected patients attending outpatient department and those hospitalized due to severe clinical signs and symptoms at country hospitals are referred to NIH for screening and confirmation.

### Sample collection and laboratory diagnosis

A total of 9 493 acute phase blood samples were analyzed for the detection of anti-dengue IgM using capture Enzyme linked immunosorbent assay (Dengue IgM ELISA, Panbio Tech Co Brisbane, Australia). Five milliliters (ml) of blood sample was drawn from suspected dengue patients attending hospitals and transported to Department of Virology, NIH Islamabad. All blood samples were centrifuged at 3000 rpm for 5 min and serum was stored at −70 °C until further processing.

### Assay procedure for the detection of anti-dengue IgM by ELISA

All serum samples were analyzed for the detection of anti-dengue IgM antibodies using IgM capture ELISA (Panbio Tech Co Brisbane, Australia) according to the instructions provided by the manufacturer. This kit is used for the qualitative detection of anti-dengue IgM antibodies in patients’ serum with reported sensitivity and specificity of 55.7–94.7% and 97–100%, respectively.

Briefly 96 well microtiter plate was precoated with anti-human IgM antibodies. 100 μl of diluted (1:100) serum sample and positive/negative controls were dispensed to the respective wells and plate was incubated at 37 °C for 1 h. During first incubation, anti-dengue IgM antibodies, if present in the patient’s serum sample were bound to anti human IgM antibodies. At the end of incubation, all wells were six times washed with wash buffer followed by addition of 100 μl diluted antigen to each well and plate was incubated at 37 °C for 1 h. During second incubation anti-dengue horse radish peroxidase conjugate was used followed by washing out the unbound antigen/antibody complex and addition of 100 μl of TMB (3, 3′, 5, 5′-Tetramethylbenzidine) substrate. The plate was finally incubated at 37 °C for 10 min. The reaction was then stopped and absorbance (optical density) was read within 30 min using as ELISA reader at a wavelength of 450 nm.

### Interpretation of assay results

The results of test were interpreted by the calculating cut-off value which is the average absorbance of triplicate of calibrator multiplied with 0.62. Index value was calculated by dividing sample´s optical density (O.D) by the cut-off value. Panbio unit was calculated by multiplying the index value with 10. The final sample results were interpreted as follow.

$$ \mathrm{Negative}\ \mathrm{Results} = \mathrm{Index}\ \mathrm{value} < 0.9\ \mathrm{and}\ \mathrm{Panbio}\ \mathrm{unit} < 9 $$

$$ \mathrm{Equivocal}\ \mathrm{Results} = \mathrm{Index}\ \mathrm{value}\ 0.9\hbox{-} 1.1\ \mathrm{and}\ \mathrm{Panbio}\ \mathrm{unit}\ 9\hbox{-} 11 $$

$$ \mathrm{Positive}\ \mathrm{Results} = \mathrm{Index}\ \mathrm{value} > 1.1\ \mathrm{and}\ \mathrm{Panbio}\ \mathrm{unit} > 11 $$

### Demographical analysis

Geographic distribution of dengue cases was determined based on the information provided on patient investigation forms. The seasonal incidence was determined by grouping all dengue cases into monthly intervals based on the time of disease onset. Medical history records of dengue cases available with the hospitals were also reviewed to collect the required data including dengue related deaths in Pakistan during 2009–2014. Data was collected from registers, patient investigation forms as well as a questionnaire was administered to obtain demographic and clinical information including the patient´s age, gender, clinical signs and symptoms, area of residence, date of admission, date of onset, travel history to dengue endemic areas, status at the time of discharge and prognosis.

The annual patient incidence (API) was calculated as the number of sero-positive (IgM+) patients per 100 000 inhabitants for each of the study sites in each year [API = (number of sero-positive patients/population) × 100 000]. Mean Annual Patient Incidence (MAPI) was calculated as- “the sum of each year’s API in the each study site divided by total study duration i.e.6 years [MAPI = (sum of 6 year’s API)/6].

### Data analysis

Data was analyzed using Statistical Package for Social Science version 20 (SPSS v.20)

## Results

From January 2009 to December 2014, a total of 9 493 blood samples from clinically suspected dengue cases were received from different districts of the country and tested for anti-dengue IgM to confirm acute dengue virus infection.

Out of 9 493 suspected cases 3 504 (36.9%) were detected positive with IgM antibodies. Among 9 493 cases, 6 858 (72.2%) were male and 2 635 (27.8%) were female. Gender wise distribution showed that 73.6% (2 578/3 504) male and 26.4% (926/3 504) female subjects were found IgM positive. Although the male to female ratio of total reported cases was 3:1, positive rate of each tested age group showed similar rates of IgM positivity between males (37.6%) and females (35.1%) (Table 1). According to the available medical records, 5.0% (472/9,493) of dengue related deaths were reported during the last six years: 2009–2014 (Table 1).

**Table 1:** Gender wise distribution of dengue cases and death rate during 2009–2014 in Pakistan

Year	Total			Male			Female			Number of patients died	Death rate (%)^e^
	No. of tested samples	No. of positive samples	Positive rate (%)	No. of tested samples	No. of positive samples	Positive rate (%)	No. of tested samples	No. of positive samples	Positive rate (%)		
2009	430	136	31.6	266	72 (52.9^a^)	27.1^b^	164	64 (47.1^c^)	39.0^d^	13	3.0
2010	3131	1303	41.6	2231	942 (72.3)	42.2	900	361 (27.7)	40.1	40	1.3
2011	4459	1477	33.1	3389	1185 (80.2)	35.0	1070	292 (19.8)	27.3	362	8.1
2012	376	20	5.3	265	16 (80.0)	6.0	111	4(20.0)	3.6	0	0.0
2013	827	356	43.0	535	228 (64.0)	42.6	292	128 (36.0)	43.8	57	6.9
2014	270	212	78.5	172	135 (63.7)	78.5	98	77 (36.3)	78.6	0	0.0
Total	9493	3504	36.9	6858	2578 (73.6)	37.6	2635	926 (26.4)	35.1	472	5.0

^a^(Male number of positive samples/Total number of positive samples) x 100

^b^(Male number of positive samples/Male number of tested samples) x 100

^c^(Female number of positive samples/Total number of positive samples) x 100

^d^(Female number of positive samples/Female number of tested samples) x 100

^e^Death rate = (Number of patients died / Total No. of tested samples) x 100

**Fig. 1:**
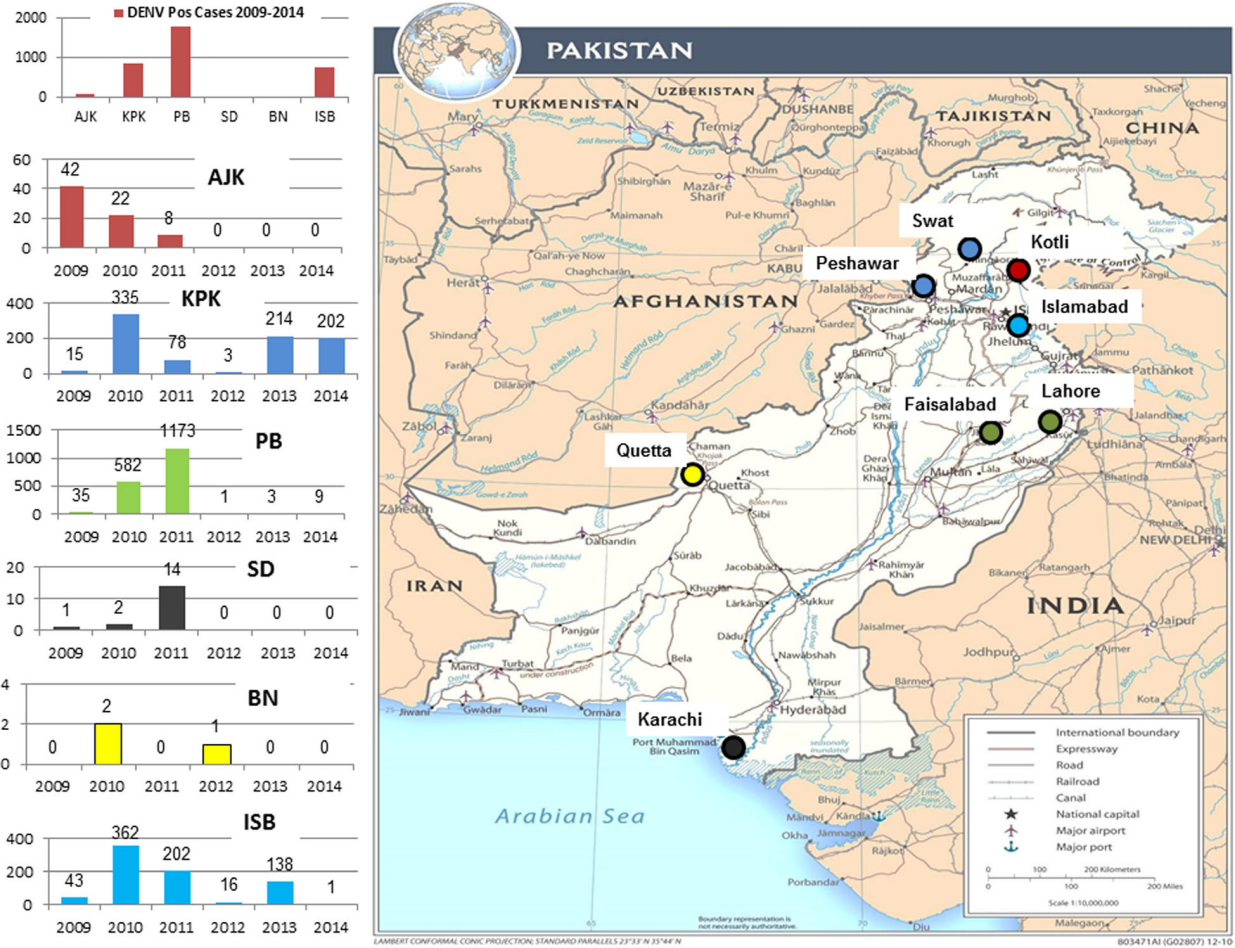
Geographical map of Pakistan showing districts with dengue postive cases during 2009-2014. The bar charts indicate number of DENV positive cases in each of the four provinces and regions of Pakistan. AJK: Azad Jammu and Kashmir; KPK: Khyber Pakhtunkhwa; PB: Punjab; SD: Sindh; BN: Baluchistan; ISB: Islamabad

Among 3 504 IgM positive cases, the highest positive rate (41.4%) was noticed among the individuals aged 31–40 years followed by those within 41–50 age groups (Table 2). The majority of positive cases (96.2%; 3 359/3 504) were detected during post-monsoon season (September-November) with only few cases (3.8%; 133/3 504) in other months, and the highest positive rate (45.7%) was detected in November (Table 3). Data presented in this study showed that the dengue fever cases were not evenly distributed throughout the country. The highest Mean Annual Patient Incidence during six years of study (2009–2014) was reported from Islamabad (11.03) followed by Khyber Pakhtunkhwa province, 0.45), Punjab province (0.33), Azad Jammu and Kashmir (0.28), Sindh province (0.005), and Balochistan province (0.003) (Fig. 1, Additional file 2: Table S1). The highest MAPI (11.03) rate reported for Islamabad is based on the overwhelmed number of dengue cases reported from its different hospitals that may be attributed to the availability of adequate medical and diagnostic services in the capital city compared to the other country hospitals.

**Table 2:** Age wise distribution of dengue cases during 2009–2014 in Pakistan

Age groups	No. of tested samples	No. of positive samples	Positive rate (%)	No. of positive samples						*P*-value
				2009	2010	2011	2012	2013	2014	
0–10	279	81	29.0	12	36	16	1	9	7	0.019*
11–20	1 230	429	34.9	34	171	79	6	72	67	
21–30	5897	2199	37.3	61	668	1249	10	155	56	
31–40	929	382	41.1	16	201	67	0	55	43	
41–50	587	238	40.5	9	127	36	2	40	24	
51–60	361	115	31.9	3	65	20	0	20	7	
>60	210	60	28.6	1	35	10	1	5	8	
Total	9493	3504	36.9	136	1303	1477	20	356	212	

*= significant *p*-value (<0.05)

**Table 3:** Month wise distribution of dengue cases during 2009–2014 in Pakistan

Month	Total no. of tested samples	No. of positive samples	Positive rate (%)	No. of positive samples					
				2009	2010	2011	2012	2013	2014
January	61	1	1.6	1	0	0	0	0	0
February	38	0	0	0	0	0	0	0	0
March	49	3	6.1	0	0	0	3	0	0
April	80	5	6.3	3	1	0	1	0	0
May	117	8	6.8	3	1	2	0	1	1
June	156	20	12.8	3	0	12	1	0	4
July	122	27	22.1	2	0	2	4	2	17
August	254	79	31.1	19	1	14	0	6	39
September	2445	741	30.3	9	15	522	6	116	73
October	4 400	1812	41.2	83	700	822	1	128	78
November	1764	806	45.7	12	585	103	3	103	0
December	7	2	28.6	1	0	0	1	0	0
Total	9493	3504	36.9	136	1303	1477	20	356	212

## Discussion

Majority of the clinically suspected cases in our study were turned out as sero-negative (63.1%, 5 989/9 493) for the dengue infection and might be hospitalized due to several related febrile illnesses. In such situation, dengue incidences are mismatched between estimated and actual number of cases and need further investigations to rule out the possibility of other mosquito-borne hemorrhagic infections especially chikungunya and yellow fever [20]. The higher incidence of infection in male subjects may reflect their higher trend of seeking medical advice and attending health units resulting in increased reporting, in contrast to females who usually prefer home remedies for cure, as evidenced in studies from India and Nepal [21]. Being a dengue endemic country, *Ae.* a*egypti* is the most prevalent dengue vector reported in urban areas of Pakistan such as Karachi, Lahore, Rawalpindi, and Attock up to haripur district but in hilly areas such as, Abbottabad, Mansehra, Swat, and Azad Jammu and Kashmir, *Ae. albopictus* had also been found during the dengue season [22]. Likewise, the dengue positive patients residing in Pakistan exhibited a wide geographic distribution, presenting four province and 99 districts.

Presence of the both DENV vector mosquitoes, *Ae. aegypti* and *Ae. albopictus* is also reported from Punjab and Khyber Pakhtunkhwa (KPK) province [23]. The recent expansion of dengue virus infection to different parts of the country may directly be linked to climatic and human factors such as increased rainfall, floods and high population density that positively enhance the vector breeding and disease transmission efficiencies.

A few published studies on dengue in Pakistan have been reported from Karachi, Lahore and Swat [7, 8, 12, 16] indicating repeated outbreaks in Karachi during 1994–2008. Karachi is the largest and most populous metropolitan city in Pakistan and considered as one of the world’s fastest growing cities attracting migrants from all over Pakistan and South Asia. Despite these facts, our data highlights Lahore as the most affected city with higher annual dengue incidence rates compared to Karachi. This is directly reflected by the smaller number of samples from Karachi referred to NIH, Islamabad for dengue diagnostics, owing to the comparable availability of medical and diagnostics services at local hospitals and medical facilities in Karachi. Dengue virus infection was first time detected in Lahore during 2006 following by a massive outbreak during 2010–2011 with ensuing transmission to many country areas. Lahore is the second largest and most populous city in Pakistan after Karachi and its higher incidence of dengue infections may be linked with the massive to and fro population movement on daily bases. Further studies are required to investigate and rule out the true disease burden in Lahore, based on the demarcation between its indigenous population and temporary visiting patients. Furthermore, during 2010 and 2011, 40 and 362 deaths were reported respectively from Punjab province [9]. During 2013–2014, sporadic DENV cases were reported from several country areas along with huge outbreak reported from Swat and Mansehra districts in KPK province resulting in 57 deaths in Swat and adjacent areas [16].

The high mortality rate during 2011–2013 in Lahore and Swat may point out multiple risk factors: a) these areas were affected by flood due to heavy rainfall which provide suitable environment for vector breeding; b) co-circulation of multiple dengue virus serotypes in these dengue endemic areas leading to significant morbidity and mortality.

In Pakistan, two standard methods, ELISA and rapid immunochromatographic test, for the diagnosis of dengue infection are available only at few major hospitals. Detection of IgM antibodies by ELISA is a sensitive marker for the identification of acute dengue virus infection. These antibodies are detectable in 50% of patients by days 3–5 after onset of illness, increasing to 80% by day 5 and 99% by day 10. In countries like Pakistan, the infected individuals’ visit the health facilities several days after the onset of disease. Considering these facts anti-dengue IgM ELISA may serve as a better diagnostic tool and be the method of choice referring to the development of IgM antibodies three to five days after the onset of clinical signs [24].

Presently, Pakistan lacks an active surveillance system for dengue and other arbovirus infections and solely rely on the passive surveillance system that does not cover the entire population, especially areas with poor health care settings. Development of an active surveillance system would enable health authorities to better monitor the disease incidence and transmission of DENV infection to identify high risk areas, and target dengue control activities.

There is no specific treatment available for dengue virus infection, so patient management is mainly supportive. Early diagnosis and mosquito vector control is the gold standard approach to control the dengue virus infection [25]. Our findings emphasize that the rapid diagnostic kits to rule out the DENV infection must be available at health facilities of known endemic areas particularly during the outbreak period (post-monsoon season) for the early diagnosis and in-time management of dengue cases [26].

## Conclusions

Incessant outbreaks of dengue infection in multiple geographical areas across Pakistan highlight the urgent need of a comprehensive surveillance and diagnostic system in order to identify the true disease burden and pinpoint risk factors. Further research on dengue virus infection in Pakistan should involve rapid diagnostic facilities, identifying circulating serotypes, studying vector population dynamics, implementation of sustainable vector control strategies, and public awareness on priority.

## Additional files

Additional file 1: Multilingual abstracts in the six official working languages of the United Nations. (PDF 388 kb)
Additional file 2: Table S1. Province wise, annual patient incidence of dengue fever cases during 2009–2014 in Pakistan. (DOCX 19 kb)

## Abbreviations

AJK: Azad Jammu & Kashmir
API: Annual patients’ incidence
DHF: Dengue hemorrhagic fever
DSS: Dengue shock syndrome
ELISA: Enzyme linked immune-sorbent assay
KPK: Khyber Pakhtunkhwa
WHO: World Health Organization
