# Supplementary material for: “Preliminary Seroepidemiological survey of dengue infections in Pakistan, 2009-2014”
Source: Infect Dis Poverty. 2017 Mar 9;6:48. doi: 10.1186/s40249-017-0258-6 (PMC5343310; doi:10.1186/s40249-017-0258-6)
Supplement: Additional file 1: — Multilingual abstracts in the six official working languages of the United Nations. (PDF 388 kb) [file 40249_2017_258_MOESM1_ESM.pdf]

Translation of the abstract into the six official working languages of the United Nations

استبيان تمهيدي للوبائيات السيرولوجية لحمى الضنك في الباكستان، 2009-2014

محمّد سليمان، هيونغ – وو لي، راني فريال1، محمّد مسرور علم، أوزما بشير عامر، نادیا ناصر، سلمان شريف، شهزاد شوكت، عدنان كرشيد، ماهر أنجز، مصعب أمير، غلما مجتبا، سعيد سهيل زاهور الزيدي

ملخّص

تقديم عام: فيروس الضنك هو العامل المسبب لحمى الضنك وهي حمى تُحمل بناقل يتسبب في مرض يهدد حياة الأشخاص. تم إجراء دراسة للوبائيات السيرولوجية بهدف فهم انتشار فيروس الضنك في الباكستان.

النتائج: تُعدّ حمى الضنك مرض مستوطن في دولة الباكستان ويشهد ارتفاعا مستمرا منذ الإعلان عن ظهوره لأول مرة في سنة 1994. لاكتشاف مدى انتشار فيروس حمى الضنك في الباكستان خلال الفترة الممتدة بين 2009 و2014، تمّ أخذ 9493 عينة من الدم للكشف عن وجود الأجسام المضادة IgM لمكافحة حمى الضنك اعتمادا على تقنية الإليزا (ELISA). تمت مراجعة المواصفات السريرية والديموغرافية المتوفرة في سجلات المستشفى للتأكد من نسبة الوفيات المرتبطة بالصدمة النزفية الناتجة عن مرض حمى الضنك. من بين 9493 عينة تم فحصها، كانت النتيجة إيجابية في ما يتعلق بوجود الأجسام المضادة IgM لمكافحة حمى الضنك بنسبة 37% (ما يُعادل 3504 عينة)، بما في ذلك 73.6% من الذكور (2578/3504) و26.4% من الإناث (926/3504). كانت أعلى نسبة إيجابية (382/929، 41.1 %) لدى الأفراد من الفئة العمرية 31-40 سنة. تم الإبلاغ عن أعلى نسبة من الحالات التي تظهر عليها أعراض المرض في أكتوبر (4400/9493، 46%) وأعلى نسبة لتأكيد الإصابة بالمرض من ضمن الحالات التي تظهر عليها أعراض المرض في نوفمبر (806/1764، 45.7%). يعني النسبة السنوية لوقوع المرض (MAPI) خلال 2009-2014 في باكستان ما تزال 0.30 وكانت أعلى نسبة للوقوع سنويا (11.03) في إسلام أباد. تمّ تسجيل 472 حالة وفاء جراء مرض حمى الضنك خلال الفترة الممتدة بين 2009 و2014 حسب سجلات المستشفيات.

الخاتمة: وصفت بيانات التقارير المنشورة سابقا في الباكستان الإصابة بحمى الضنك في المناطق المحدودة من البلاد. إن نتائج دراستنا مهمة في ما يخصّ اختبار العينات السريرية لنسبة أعلى من المرضى على نطاق أوسع وتنفيذ الأوامر المتعلقة ببرامج مراقبة نواقل حمى الضنك ومكافحتها بشكل مناسب.

Translated from English version into Arabic by Zeineb TRABELSI, through

TRANSLATORS WITHOUT BORDERS

200-2014 年巴基斯坦登革热血清流行病学调查

Muhammad Suleman, Hyeong-Woo Lee, Rani Faryal1, Muhammad Masroor Alam, Uzma Bashir Aamir, Nadia Nasir, Salmaan Sharif, Shahzad Shaukat, Adnan Khurshid, Mehar Angez, Massab Umair, Ghulam Mujtaba, Syed Sohail Zahoor Zaidi

摘要

引言：登革热是一种媒传疾病，危及人类生命，其病原体是登革病毒。在巴基斯坦对登革热病毒进行血清流行病学研究以了解其当前流行病学特征。

结果：巴基斯坦是一个登革热流行国家，1994 年首次报告登革热暴发，随后其发病率持续上升。我们使用 ELISA 共筛选 9 493 份血样，检测抗登革热 IgM 抗体，研究 2009-2014 年间巴基斯坦登革热的流行率。审查了医院记录可用的临床和人口统计学特征，以确定与登革出血休克综合征相关的死亡率。在受测的 9 493 个样本中，37%（3 504）抗登革热 IgM 抗体呈阳性。在血清阳性病例中，73.6%（2 578/3 504）是男性，26.4%（926/3504）是女性。31-40 岁年龄组血清阳性病例数最高（382/929; 41.1%）。10 月份报告的有症状病例数最高（46%; 4

400/9 493），11 月有症状病例中血清阳性病例数最高（45.7%; 806/1 764）。2009-2014 年，年平均患者发病率（MAPI）仍为 0.30/10 万，伊斯兰堡的年发病率最高，达 11.03/10 万。根据医院记录，2009 至 2014 年报告的登革热相关死亡人数为 472。

结论：之前巴基斯坦公开发表的报告数据描述了该国某些地区的登革病毒感染率。我们的研究覆盖广大区域对大规模患者的临床样本进行测试是非常重要的，并保证及时实施登革热媒介监测和控制计划。

Translated from English version into Chinese by Jin Chen, edited by Pin Yang

CHINESE CENTER FOR DISEASE CONTROL AND PREVENTION
NATIONAL INSTITUTE OF PARASITIC DISEASES

Étude séro-épidémiologique préliminaire de la dengue au Pakistan, 2009-2014

Muhammad Suleman, Hyeong-Woo Lee, Rani Faryal1, Muhammad Masroor Alam, Uzma Bashir Aamir, Nadia Nasir, Salmaan Sharif, Shahzad Shaukat, Adnan Khurshid, Mehar Angez, Massab Umair, Ghulam Mujtaba, Syed Sohail Zahoor Zaidi

Résumé

Introduction: le virus de la dengue est à l’origine de la dengue ou « grippe tropicale », une infection à transmission vectorielle. Chez l’homme, la maladie qui en résulte peut être spontanément résolutive mais aussi évoluer vers une forme engageant le pronostic vital. Une étude séro-épidémiologique a été réalisée afin de comprendre l’épidémiologie actuelle du virus de la dengue au Pakistan.

Résultats: le Pakistan est un pays où la dengue est endémique et où le nombre de cas augmente régulièrement depuis la première flambée rapportée en 1994. Pour étudier la prévalence du virus de la dengue au Pakistan au cours de la période 2009-2014, des échantillons sanguins, au nombre de 9493, ont été analysés à l’aide d’une méthode ELISA permettant la détection des anticorps IgM dirigés contre le virus de la dengue. Le nombre de décès dus à une dengue hémorragique avec syndrome de choc a été établi sur la base des données cliniques et démographiques tirées des dossiers hospitaliers. Sur les 9 493 échantillons analysés, 37 % (3 504) étaient positifs en termes d’anticorps IgM dirigés contre le virus de la dengue. Les hommes représentaient 73,6 % (2 578/3 504) des cas séropositifs et les femmes 26,4 % (926/3 504). Le plus grand nombre de cas séropositifs (382/929; 41,1 %) était observé dans la tranche d'âge comprise entre 31 et 40 ans. Le nombre le plus important de cas symptomatiques était signalé en octobre (46 %; 4 400/9 493) et le nombre le plus élevé de cas séropositifs parmi les cas symptomatiques concernait le mois de novembre (45,7 %; 806/1 764). L’incidence annuelle moyenne est restée stable, à 0,30, au cours de la période 2009-2014 au Pakistan, l’incidence annuelle la plus forte (11,03) étant observée à Islamabad. D’après les dossiers hospitaliers, le nombre de décès liés à la dengue a été de 472 au cours de la période 2009-2014.

Conclusion: les données issues des articles publiés précédemment sur les cas de dengue au Pakistan concernaient l’incidence du virus dans des régions limitées du pays. Nos résultats sont importants car l’analyse de notre étude a été réalisée à plus grande échelle, les échantillons cliniques de patients provenant de régions géographiques étendues. Ces résultats justifient la mise en place rapide de programmes de surveillance des vecteurs de la dengue et de lutte anti-vectorielle.

Translated from English version into French by Véronique Nowak-Solinska, through

Предварительное сероэпидемиологическое исследование инфекции Денге в Пакистане, 2009-2014

Мухаммад Сулеман, Хиеонг-Ву Ли, Рани Фарьял, Мухаммад Масроор Алам, Узма Башир Аамир, Надя Назир, Салмаан Шариф, Шахзад Шаукат, Аднан, Хуршид, Мехар Ангез, Массаб Умаир, Гулам Муджаба, Сайед Сохаил Захоор Заиди

Краткий обзор

Исходные данные: Вирус денге - это возбудитель лихорадки денге, трансмиссивной инфекции, вызывающей у людей болезнь, которая может пройти сама по себе либо стать угрозой для жизни. Сероэпидемиологическое исследование было произведено для того, чтобы понять текущую эпидемиологию вируса денге в Пакистане.

Полученные данные: Пакистан является страной, в которой вирус денге широко распространен, и охваченная им территория постоянно увеличивается со времени первой вспышки в 1994 году. Для изучения распространенности вируса денге в Пакистане в 2009-2014 годах было проведено скрининговое исследование 9 493 образцов крови на обнаружение IgM антител к денге при помощи иммуно-ферментного анализа. Были просмотрены клинические и демографические характеристики, имеющиеся в больничных записях, чтобы установить коэффициент смертности, относящийся к шоку при геморрагической лихорадке денге. Из 9 493 протестированный образцов 37% (3 504) были положительны на IgM антитела к денге. Из серopоложительный случаев 73,6% (2 578/3 504) - мужчины, и 26,4% (926/3 504) - женщины. Самое высокое число (382/929; 41.1%) серopоложительных случаев наблюдались у пациентов возрастной группы 31-40 лет. Наивысшее количество симптоматических случаев было зарегистрировано в октябре (46%; 4 400/9 493), а наивысшее количество серopоложительных случаев среди симптоматических было в ноябре (45.7%; 806/1 764). Среднегодовой уровень заболеваемости в течение 2009-2014 г.г. в Пакистане оставался в пределах 0,30, с наиболее высоким годовым уровнем заболеваемости (11,03) зарегистрированным в Исламобаде. . В соответствии с больничными записями, в течение 2009-2014 г. зафиксировано 472 летальных исхода связанных с этим заболеванием.

Заключение: Данные, полученные от ранее опубликованных отчетов в Пакистане, описывают распространенность вируса денге из ограниченных областей страны. Наши полученные данные важны, поскольку они получены от тестирования клинических образцов в крупных масштабах от пациентов в широких географических регионах, и они обеспечивают своевременное внедрение эпидемиологического надзора и программ контроля за переносом вируса денге.

Translated from English version into Russian by Natallia Lupik, through

Encuesta seroepidemiológica preliminar sobre infecciones por dengue en Pakistán, 2009-2014

Muhammad Suleman, Hyeong-Woo Lee, Rani Faryal1, Muhammad Masroor Alam, Uzma Bashir Aamir, Nadia Nasir, Salmaan Sharif, Shahzad Shaukat, Adnan Khurshid, Mehar Angez, Massab Umair, Ghulam Mujtaba, Syed Sohail Zahoor Zaidi

Resumen

Antecedentes: El virus del dengue es el agente causante de la fiebre del dengue, una infección de transmisión vectorial que causa desde enfermedades autolimitadas a enfermedades mortales en humanos. Se ha llevado a cabo un estudio seroepidemiológico para entender la epidemiología actual del virus del dengue en Pakistán.

Hallazgos: Pakistán es un país con dengue endémico que ha tenido un ascenso constante en su incidencia desde que se declaró un brote por primera vez en 1994. Para investigar la persistencia del virus del dengue en Pakistán entre 2009 y 2014, se examinaron 9 493 muestras de sangre para detectar anticuerpos IgM específicos al dengue usando ELISA. Se revisaron los síntomas clínicos y características demográficas disponibles en historiales médicos para confirmar las defunciones relacionadas con el síndrome de shock por dengue hemorrágico (dengue grave). De las 9 493 muestras analizadas, el 37% (3 504) dieron positivo por anticuerpos IgM específicos al dengue. De los casos seropositivos, el 73,6% (2 578 de 3 504) eran varones y el 26,4% (926 de 3 504) eran mujeres. Se observó el número más elevado de casos seropositivos (382 de 929; el 41,1%) en individuos de entre 31 y 40 años. Se registró el número más elevado de casos sintomáticos en octubre (46%; 4 400 de 9 493) y el número más elevado de casos seropositivos entre los casos sintomáticos ocurrió en noviembre (45,7%; 806 de 1 764). La incedencia de pacientes media anual entre 2009 y 2014 en Pakistán se quedó en 0,30 y la incidencia de pacientes anual más alta (11,03) se halló en Islamabad. Según los informes médicos, se registraron 472 muertes relacionadas al dengue entre 2009 y 2014.

Conclusiones: Los datos incluidos en informes anterioremente publicados en Pakistán describían la incidencia del virus del dengue en zonas limitadas del país. Nuestros resultados son importantes si se tiene en cuenta que se han analizado muestras clínicas a una escala mayor que han cubierto vastas regiones geográficas y que se garantiza una puesta en marcha a tiempo de la vigilancia del vector del dengue y programas de control.

Translated from English version into Spanish by Carmen Sanchez-Navarro, through

TRANSLATORS
WITHOUT BORDERS
