# Supplementary material for: “Preliminary Seroepidemiological survey of dengue infections in Pakistan, 2009-2014”
Source: Infect Dis Poverty. 2017 Mar 9;6:48. doi: 10.1186/s40249-017-0258-6 (PMC5343310; doi:10.1186/s40249-017-0258-6)
Supplement: Additional file 2: Table S1. — Province wise, annual patient incidence of dengue fever cases during 2009–2014 in Pakistan. (DOCX 19 kb) [file 40249_2017_258_MOESM2_ESM.docx]

| **Table S1. Annual patient incidence of dengue cases during 2009-2014 in Pakistan** | | | | | | | | | | | | | | | |
| --- | --- | --- | --- | --- | --- | --- | --- | --- | --- | --- | --- | --- | --- | --- | --- |
| **Province** | **Population** | **No. of Positive** | **API* (MAPI^††^)** | **2009** | | **2010** | | **2011** | | **2012** | | **2013** | | **2014** | |
|  |  |  |  | **No. of Positive** | **API** | **No. of Positive** | **API** | **No. of Positive** | **API** | **No. of Positive** | **API** | **No. of Positive** | **API** | **No. of Positive** | **API** |
| **AJK^†^** | 4,256,617 | 72 | 1.69 (0.28) | 42 | 0.99 | 22 | 0.52 | 8 | 0.19 | 0 | 0.00 | 0 | 0.00 | 0 | 0.00 |
| **KPK^**^** | 31,349,742 | 847 | 2.70 (0.45) | 15 | 0.05 | 335 | 1.07 | 78 | 0.25 | 3 | 0.01 | 214 | 0.68 | 202 | 0.64 |
| **Punjab** | 91,379,615 | 1,803 | 1.97 (0.33) | 35 | 0.04 | 582 | 0.64 | 1,173 | 1.28 | 1 | 0.00 | 3 | 0.00 | 9 | 0.01 |
| **Sindh** | 55,245,497 | 17 | 0.03 (0.005) | 1 | 0.00 | 2 | 0.00 | 14 | 0.03 | 0 | 0.00 | 0 | 0.00 | 0 | 0.00 |
| **Balochistan** | 13,162,222 | 3 | 0.02 (0.003) | 0 | 0.00 | 0 | 0.00 | 2 | 0.02 | 0 | 0.00 | 1 | 0.01 | 0 | 0.00 |
| **Islamabad** | 1,151,868 | 762 | 66.15 (11.03) | 43 | 3.74 | 362 | 31.43 | 202 | 17.54 | 16 | 1.39 | 138 | 11.98 | 1 | 0.09 |
| **Total** | **196,545,561** | **3,504** | **1.78** | **136** | **0.07** | **1,303** | **0.66** | **1,477** | **0.75** | **20** | **0.01** | **356** | **0.18** | **212** | **0.11** |

* Annual Patient Incidence = (No. of sero-positive/Population) x 100,000

†† Mean Annual Patient Incidence = [ (No. of sero-positive/Population) x 100,000]/6 years

**†**Azad Jammu & Kashmir

**Khyber Pakhtunkhwa
